# Bibliographical Notices

**Published:** 1856-11

**Authors:** 


### BIBLIOGRAPHICAL NOTICES.

*Digestion and its Derangements: The Principles of Rational Medicine applied to Disorders of the Alimentary Canal.* By THOMAS K. CHAMBERS, M. D., Fellow of the College of Physicians; Physician to St. Mary's Hospital, and Lecturer on the Practice of Medicine at St. Mary's Medical School, London, &c. New York, S. S. & W. Wood, 261 Pearl Street: 1856: 8vo. pp. 441.

Seldom have we been called upon to peruse a work so full of interest to the physiologist and the daily practitioner as the one now before us. Dr. Chambers already favorably known as the author of *Decennium Pathologicum*, of the Gulstonian Lectures on *Corpulence, or Excess of Fat in the Human Body*, and of various detached articles on *Dietetics, Microscopic Anatomy, &c.*, has certainly imposed an obligation on the profession, by the publication of this laborious and useful "attempt to join the *disjecta membra* of recent observations on the subject of digestion into a connected sketch—to offer a concise picture of these vital processes, such as may be present to the mind of a practitioner, who is continuously employed in modifying them for the relief of pain and preservation of life, without overloading his scanty leisure by prolonged quotation."

The growing importance of physiology none can deny. Through long years of patient and unwearied toil, it has struggled, on, unhesitatingly asserting, on all proper occasions, its high claims to respectful consideration as the able comate of pathology and the help-meet of the scientific and therefore "regular" physician. The enemies of physiology formerly denied its utility, and assiduously attempted a display of the many shortcomings and inaccuracies necessarily incident to its youth; these efforts having proved unavailing, it has now become fashionable with some to discredit its applicability to practical medicine, and to rely wholly upon the observation of morbid phenomena for the principles which are to guide them in the eradication of disease. "To those," writes Renouard, in his *History of Medicine*, "who pretend to deduce the general rules of treatment from some opinion, or physiological experiment, we would recall the axiom of philosophy already invoked by us more than once: In the succession of natural phenomena, nothing presents to us the idea of causality, or the necessary relation of cause and effect. * * * * When the cure of an order of diseases follows constantly the employment of a medication, we are led to regard this medication as the cause of the cure which follows its use; *but it is impossible for us to perceive the physiological reason of this result, and it is consequently useless to seek it.* * * * * The physiologist must limit himself to the description of the normal phenomena of the living economy—the pathologist to abnormal ones."* If this be true, then should the profession at once close ranks with the army of quacks, and abandoning all claim to science and philosophy, march on, or rather retrograde gropingly, to the sublime and ambitious strain of *Experentia docet.* A careful consideration of the manner in which the symptoms of a given disease vary in different individuals, in accordance with certain slight and scarcely appreciable peculiarities of structure, is sufficient to show that post-mortem examinations and the observations of diseased actions, no matter how carefully conducted they may be, cannot, of themselves lead to that accuracy of diagnosis and precision of treatment, which alone, in virtue of its successful results, is capable of commanding the respect and allegiance of the people for legitimate medicine. Healthy structure and healthy life functions must first be studied, ere we can understandingly proceed to the investigation of diseased tissues and their abnormal manifestations. Through some defect of the nutritive or absorption processes, an organ undergoes structural change; sooner or later altered function ensues, which, in its turn, produces a molecular change in some neighboring organ; death finally terminates the series and the pathologist is called upon to discriminate between cause and effect, between primary and secondary conditions, between the starting point and the terminus of the fatal malady. How vain the attempt unless aided by the auxiliaries above indicated. As in the mineral, the vegetable and animal kingdoms, the naturalist is constantly called upon to witness the almost imperceptible steps by which the distinguishing characters of the objects of

* History of Medicine from its origin to the nineteenth century, &c. By P. V. Renouard, M. D. Translated by C. G. Comegys, M. D.

his research pass into and blend with each other so effectually as to break down all the arbitrary distinctions of species, &c., so the observant physician has daily opportunities of perceiving how healthy tissues and functions pass by such insensible degrees into diseased structure and actions that no positive line of demarcation can be drawn between them. This fundamental and significant fact alone, should lead us to recognise in the laws of physiology the *punctum saliens* of all pathologic phenomena. Indeed, theoretical and practical medicine can never become a rational, philosophical system, unless it be based upon a strictly scientific physiology, whose numerous and complicated phenomena have been carefully generalized in accordance with the inductive method, and reduced to a few simple laws and expressions.

Every practical physician is painfully aware that the popular mind entertains but one criterion of his intelligence and skill. This criterion is his success in ameliorating the sufferings of his patients, and restoring them to their wonted health. So that he escapes his dreaded disease, the man with the dyspeptic stomach or tuberculous lung, cares but little for the manner in which his relief was effected. Rejoicing in his renewed health, he leaves the *methodus medendi* to his medical attendant. But in this method lie the *principles* of our art, which are carefully to be distinguished from its *agents*,—the drugs and mechanical appliances upon which the empiric and the routinist wholly depend for that success which is inseparably connected with the method, rather than with the drug; with the principle, rather than with the agent. Let the physician, therefore, be less anxious about the empirical administration of the materia medica, and more mindful of the Institutes of the healing art, those laws which in the aggregate constitute what may be called the corpus medicinæ, comprising as they do both the healthy and diseased actions of the economy, and leading to an accurate system of diagnosis and therapeutics, the only true foundation of continued success in the management of disease. Only by the philosophical cultivation of the principles of medicine, can the profession, as a body, hope to obtain that decided and constant success in the treatment of disease, which will alone effectually separate them, in the eyes of the people, from the fraternity of quacks, who, from the time of the venerable Hippocrates himself, down to the present moment, have clung with such vampire-like tenacity to the body of the healing art.

The sick, in general, propound but two leading and anxious questions to their medical attendants:—What is the nature of my disease? and what drug must I swallow to be cured? The physician may evade the first, as is done every day by some theory concerning blood and nerve and muscle,—a theory oftentimes more remarkable for the unintelligible language in which it is clothed, than from any inherent profundity,—and thus cajole his patient into admiration of his learning. But in answer to the second question, a practically successful response is imperatively demanded. The inexorable patient will not be satisfied with anything short of an effective remedy. Failing this, in consequence of an inadequate acquaintance with the elementary laws of health and disease, and an overweening confidence in drugs manifested by his medical attendant, he seeks the advice of another, and another physician, and at last falls into the hands of some charlatan, who, in consequence of a mere guess or lucky coincidence is loaded with the indiscriminate praises of the self-deceived patient. Now this humiliating spectacle will continue a matter of every day occurrence, so long as physicians rely upon the observation of disease alone, without ascertaining its relations to the healthy functions. The routinists who have certain remedies for each and every affection, and who conscientiously believe that drugs "cure" disease, are perhaps more dangerous to regular medicine, than the open quack who professes to cure all maladies with his one infallible panacea.

Never has medicine needed a strictly philosophical culture so much as at the present day, when observations and experiments have been multiplied almost to infinity, and narrow and conflicting theories,—framed to account for small and detached groups of phenomena,—are usurping the place in the medical mind which should be occupied by broad and comprehensive principles. But physiology is the hand-maid of medicine, and to physiology especially, should the laws of severe, critical philosophy be applied, otherwise the nosologist, the therapeutist, and the pathologist can have no normal, well-established principles to which to refer, and from which to reason, concerning the classification and laws of disease and the remedial agency of drugs.

Entertaining such views we cordially agree with the opinions expressed by our author in the following paragraph, taken from the introduction to his work.

"The author believes that in no way is both the science and the art of healing so likely to be improved, as by the association in its literature, and through that, in the minds of its practitioners, of pathology with physiology, rather than with morbid anatomy; that juster theoretical views are elicited by looking upon disease as part of the phenomena of life, than as the producer of appearances seen after death; and that patients are more likely to be cured by one, whether original observer or reader, who is considering even imperfectly the vital actions exhibited by them, than if he knew exactly what would be the consequences of the disease in the corpse." (p. 14.)

Unlike the work of Dr. Budd, *On the Organic Diseases and Functual Disorders of the Stomach*, noticed by us in a former number of this Journal, (March, 1856,) the present volume is divided into two parts, of which the first is entirely occupied with a description of the microscopic anatomy and physiology of the digestive apparatus, carefully condensed from the published works of Kölliker, Bidder and Schmidt, Bernard, Frerichs, Lehmann, and other celebrated histologists and experimental chemists and physiologists of the present day. In the second part, the various derangements of the digestive organs are considered in their relations to the physiological laws and principles advocated in the first book. Although the author modestly, and appropriately disclaims any originality for the physiological part of his work, yet he must, by no means, be looked upon as a mere compiler, for he has not only brought together, in a condensed form, the most recent and the most reliable facts concerning the digestive act, but has also interspersed them with judicious remarks and suggestions, and demonstrated the value of not a few isolated facts, by applying them to the alleviation of human misery and suffering. The opinions inculcated are comprehensive, and in several instances assume quite a philosophical character.

Dr. Chambers' style of composition compares rather unfavorably with his matter. Though concise and often energetic, his language is not unfrequently wanting in polish and clearness, in consequence of certain badly selected terms, in which his meaning is conveyed, and the occasional tendency towards involved and complicated sentences. These slight blemishes will no doubt be corrected in a second edition, which we venture to predict will soon be called for by the medical public. The American reprint of the Messrs. Wood is very reprehensible for the orthographic blunders which disfigure it in many places, and which a little care on the part of the *employés* of the printing office might easily have avoided. If our publishers must borrow from a foreign press, let them at least do it in a respectable manner. In all other respects, the "getting up" of the work is creditable.

With these preliminary remarks, we proceed at once to lay before our readers a brief analysis of the contents of the volume under consideration.

In Chapter I. the author treats us to an interesting anatomico-physiological *resumé* of the "parts of the organism concerned in digestion, which are common to the whole alimentary canal," dwelling in succession upon the epithelium, the blood-vessels, and blood, the mucus, water, nerves, muscles and media of solution. These solid and fluid parts are discussed more in their physiological bearings, and with the ultimate view of applying the laws of their healthy functional condition to those deranged actions of the economy which in the aggregate we term disease. The structure of the epithelium of the alimentary canal, is first examined, and compared with that of the epidermis. Not only in the form of their ultimate particles are these two delicate tegumentary tissues alike, but also in chemical constitution and in their behavior when subjected to different chemical reagents, as shown by Dr. Tilanus of Arnheim, who has carefully studied the effects produced by the application of dilute and concentrated caustic potash, acetic and nitric acids, ferrocyanuret of potassium, &c., to these tissues. The difference between them is a physical one, dependent upon the different arrangement of the cells and cell-nuclei.

"We thus get an idea of a surface composed of numberless similar bodies; in the first place, *separate individuals*, and therefore with intervals between them; secondly, *not easily destroyed by chemical or* (from their minuteness) *by mechanical agents*, and therefore peculiarly permanent; thirdly, much *modified, temporarily*, in their shape by these agents, especially by the most common of them—water; admitting the substances in different degrees quickly and freely, parting with them when subjected to various processes with equal facility." (p. 18.)

The blood-vessels are next examined, and an attempt made to set forth the relation between the perfection and rapidity of the circulation on one hand, and the absorbing power of the mucous membrane on the other. After alluding briefly to the well-known laws of endosmosis established by the labors of Fischer, Dutrochet, Poisseule, Graham, Kürschner, Jolly, &c., Dr. C. says:

"There are, then, in the circumstances under which the blood-vessels and the contents of the bowels are placed, three very marked principal things, which promote the passage of fluids into the former from the latter in a greater degree than the reverse. These are: 1. The comparative density of the blood; 2. Its rapid motion; 3. Its alkalinity. While at the same time the animal warmth keeps up the general activity of the endosmosis in both directions, and probably certain uninvestigated peculiarities of the membrane lend their aid."

The three conditions here indicated are constantly varying ones. The very act of endosmosis serves to diminish the density of the blood; while, on the other hand, the action of the cutaneous surface, the lungs and the kidneys, increases this density. Every variation in the amount of moisture in the atmosphere must produce some alteration in the specific gravity of the blood, by accelerating or retarding the evaporating process. The habit of taking large or small quantities of fluid, and diseases affecting the above mentioned surfaces, must all give rise to variations in the blood. But in addition to this fluctuation in the absolute density of the blood, there is also a relative variation in the density of this fluid in different parts of the body. Dr. Schmidt always found the blood of the vena portæ of less specific gravity than that of the jugular vein. The difference was most evident after copious draughts of water, and very slight after a full, rich meal. The absorptive process is also constantly affected by changes in the motion of the blood. Influenced as the blood is by the nervous system, by its own density, by varying barometric pressures, by the effects of disease, &c., this motion must necessarily be very fluctuating. The alkalinity of the blood is another varying element affecting the process of absorption. Drs. Bidder and Schmidt have calculated that the daily secretion of bile, saliva, pancreatic and intestinal fluids, deprives the blood of a quantity of soda, one half greater than that contained in it at any one time. Under ordinary circumstances, a large proportion of this soda again finds its way into the blood. It is evident from this statement that all the conditions which modify the secretions must affect the alkalinity of the blood and thus indirectly the absorptive function. The use of salt in large quantities increases the alkalinity of the blood ; the effect of bleeding, as Dr. Zimmermann has shown, is to diminish the alkalies both absolutely and in relation to the neutral chlorides. "The diminution and increase of the alkaline salts seem to run very much hand in hand with the diminution and increase of the globules in artificially induced conditions."

Another interesting point connected with endosmosis as it goes on in the alimentary canal, is the fact, first noticed by Œsterlen, that finely divided solids are capable of passing through the coats of the vessels. The experiments of Œsterlen, Eberhard, Mensonides and Donders have shown that charcoal, sulphur, starch, quicksilver, &c., were capable of this sort of solid endosmosis. Such are some of the facts which go to show the relation of the blood-stream to absorption.

Dr. Chambers devotes several pages to the consideration of mucus—a tenacious, slippery and elastic fluid covering the delicate epithelium, and protecting it from mechanical and chemical injury.

"The great quantity of water it is capable of retaining, by swelling up, assists in keeping constantly damp the membranes over which it is spread. Thus is fulfilled one of the first essentials to the process of endosmosis or absorption. There is another use of mucus, which is not indeed connected with digestion, and perhaps therefore is a little out of place here, but which I should like to mention, because it appears to me very important, and I do not find it usually thought of. The membranes which furnish this secretion seem to be placed as *safety valves to morbid action.* A man gets his feet wet, and in consequence an uncomfortable state of blood is produced, which he feels all over: a good cold in the head comes out and sets things to rights. Or he absorbs the poison of cholera from air or water,—a diarrhœa arises, which, if the patient's strength can bear the remedy, effects the cure. Worse things happen if those natural terminations are prevented. Inflammation of bone is a terrible disorder, so in parts of the osseous system ill-defended from changes in temperature and other causes of injury, cavities lined with mucous membrane are provided, in which temporary morbid action can occur without any very serious inconvenience. Such are the frontal and neighboring sinuses, the cavity of the tympanum, and in a minor degree the synovial sacs of the joints. The intersititial inflammation of visceral glands which is so likely to end in abscess, such for example as of the liver or kidneys, may be often prevented by purulent secretion at their outlets, and the proneness of the bowels to diarrhœa will account for the infrequency of peritonitis. We may discover the reason for the frequency with which the mucous membranes are made the outlets for morbid action, in a very slight comparative injury which it there produces. In a catarrh the capillary bloodvessels become distended and clogged up without much pain, mucous globules are poured out in excessive numbers, fibrine forms, and is washed away in small flakes, pus also mixes with the mucus, and is easily diffused through the copious stream of water which is pouring by, without any harm to the surrounding textures at the time, or any permanent alteration of tissue. Whilst if the same series of actions are gone through anywhere else, we have racking pains, fibro-purulent deposits, abscesses, and fibrinous cicatrices are left behind when all is over. The wise physician imitates nature in this particular as in many others, and does not mind producing all sorts of disturbances in the mucous membranes by means of his drugs, if by so doing he can prevent other injury. He knows that the mischief he makes will soon cure itself, while that which he stops is dangerous to life or comfort. The idea of the effect of treatment on the mucous membranes cannot be too often presented to the mind. It may serve as a counterpoise to that delusion even now so prevalent among medical men, of calculating on the action of medicines only in so far as they are absorbed in the blood. I have no doubt on my own mind, not only that many substances have through the mucous membranes a powerful influence over the constitution without being dissolved in the blood at all, but that many of those which are so dissolved still owe their chief strength to their effect on the tissue we are speaking of."

Among other startling facts, the labors of modern chemistry have made us aware of an extraordinary aqueous circulation which is constantly going on through the alimentary mucous membrane, dissolving and transporting substances into the inmost recesses of the body, and carrying others out into the stomach and bowels. Some idea of the amount of this aqueous fluid is obtained when we reflect that a healthy man, weighing about 140 pounds, secretes daily from twenty-one to twenty-five pounds of bile, gastric, pancreatic and intestinal juices, of which quantity about 96 per cent. is water.

The next section treats of the physiology of the nerves of the alimentary canal. According to Bidder and Schmidt, the celebrated experimental physiologists of Dorpat—

"The division of the vagus nerve in no wise takes away the sensation of hunger. Of the food and drink taken, however, but little if any arrived at the stomach, as was easily seen through the fistulous hole. On the contrary, the matters swallowed were, even during the time of eating and drinking, generally returned by vomiting; so that it often happened that animals, without doubt pressed by hunger and thirst, repeated on the matters thrown up the operation of swallowing as many as fifteen or sixteen times, till they were quite tired and gave it up to begin again after a longer or shorter pause. All those symptoms seemed to us, without any doubt, to depend on the paralysis of the lower part of the gullet, arising out of the division of the vagus nerve in the neck. The stomach certainly had nothing to do with them, for the matters vomited had none of the acid reaction which would have distinguished them under those circumstances."

The effects of the division of this nerve on the physiological action of the gastric juice, deducted from a series of experiments upon animals by the same experimentalists, are also given. Their conclusions are,—

"That the solvent powers of the stomach on albumen taken as food are not annulled by breaking the connexion of the pneumogastric nerve in the neck, but that still it is considerably diminished in rapidity.

'This latter result may with good reason be attributed to the lessened proportion of the secretion, which, if not indirectly caused by the shock of the operation, and an unnecessary consequence thereof, yet is the only change in the activity of the stomach which arises from the division of the vagi nerve.'"

After noticing certain differences of nervous action, manifested in various localities of the alimentary canal, our author glances briefly at the muscular structure of this tract, and concludes the chapter with some remarks upon the few positive results which have hitherto attended the attempted application of analytical chemistry to physiology.

Chapter III is occupied with the consideration of the epithelium, blood vessels and secerning glands of the mouth and œsophagus, and the formation, composition, physical properties and physiological actions of the saliva. It is well-known that the quantity of saliva secreted in twenty-four hours is constantly varying. It is most abundant during the day, and is increased by the mastication of hard and stimulating substances, by picking the teeth, by the manipulations of the dentist and by mental emotions. Its minimum is attained during the night. The amount of saliva secreted by a man of ten stone weight, is estimated by Bidder and Schmidt at about three pounds per day of twenty-four hours.

After a meal, large quantities continue to be secreted and swallowed, with a view probably to the ultimate solution of alimentary bolus in the stomach. Whether secreted slowly or rapidly, in small or large quantities, the quality is invariably the same. The only difference in composition is the constant one between the fluids of the different salivary glands. So much water enters into the composition of the parotid secretion, that Bernard, Bidder and Schmidt regard these organs as *glandes aquipares* only. In 1000 parts of the parotid fluid, only 4·7 are solid, while the mixed secretion of all the glands, together with the buccal mucus contain 10·37 parts.

The conversion of amylaceous matters into sugar, by contact with saliva, has recently exercised the attention and intelligent study of Messrs. Bidder and Schmidt, who particularly dwell upon the rapidity of this change as a test to distinguish this conversion from that produced by other secretions, both healthy and morbid.

From the researches of Drs. Bidder, Schmidt and Jacubowitsch it appears that to none of the secretions alone, which, by their union constitute saliva, can be attributed the power of converting starch into sugar, but only to a certain admixture of them; that the converting power of the saliva resides in the buccal mucus mixed with the sub-maxillary fluid; that the buccal mucous membrane of young animals is devoid of action upon starch; and that in infancy the converting power is feeble.

Notwithstanding the numerous experiments instituted with the greatest skill, and the most untiring zeal, the whole subject of the saccharine conversion of starch is involved in much doubt. Thus we are by no means positive whether the converting action is entirely arrested in the stomach or not. That it is decidedly impeded is pretty well ascertained. What the impediment is has yet to be demonstrated. It has been ascribed to the *acidity* of the gastric fluids. However this may be, the arrest appears to be temporary, and, according to Bidder and Schmidt, is closely proportioned to the degree of acidity. Experiments are still wanting to determine whether the action again commences in the alkaline intestines beyond the pyloric orifice, and even whether it does not to some extent go on in the stomach during some alkaline intervals. The converting power of the saliva is impeded by the gastric juice in some animals more than in others. Thus, Dr. Jacubowitsch found, that the saliva of dogs mixed with gastric juice acted very slowly upon starch, while human saliva mixed with the same gastric fluid acted eight times more rapidly. A solution of starch injected through a fistulous orifice into the stomach of a woman fasting, yielded a large quantity of sugar in a very few minutes. Comparing such facts with the results of a number of experiments by Drs. Bidder and Schmidt upon different animals, Dr. Chambers is inclined to conclude, "that in our race gastric juice arrests much less completely than in dogs the powers of the salivary secretion, and that *by reason of the greater powers of the latter, and not necessarily any less powers resident in the former.*" This somewhat novel point is well worth the attention of experimenters.

Strange as it may appear, it is still a disputed question whether the sugar obtained from starch is to be found in the stomach or not. If it exists there, why is it not always to be found? if not, what becomes of it? What other changes does it undergo? What are its uses, &c.? The experimental physiologists of the day are not agreed upon these questions.

"In fine, the present state of our knowledge seems to render it probable that *of boiled or disintegrated starchy food, a considerable portion is turned into sugar by the saliva in its way to, and during its stay in the stomach; but that in the stomach the conversion is delayed, to be again renewed in the duodenum; that a good deal of the sugar is turned into lactic acid, and some absorbed in one form or the other.*" (P. 69.)

In Chapter IV we meet with a description of the minute anatomy of the blood-vessels, glands, epithelial lining and lymphatics of the stomach, and an account of the appearance and composition of the gastric juice.

Few questions in physiology, have commanded more attention, been more assiduously examined or more learnedly discussed, than the *acidity of the gastric juice and its cause.* Except under certain disturbing circumstances, with some of which we are acquainted, the gastric juice is known to be always acid in its reaction. An excess of mucous secretion, mental annoyance, and long rest and abstinence give to this fluid an alkaline reaction. A peculiar and as yet unexplained variation in the degree of acidity of the gastric juice has been frequently observed. That for healthy digestion an excess of acid appears to be necessary, is pretty well settled. But what is the acid whose presence is so essential to the solution of aliment. Drs. Bidder and Schmidt, as the result of eighteen experiments upon the gastric juice of dogs, who were made to fast from eighteen to twenty hours, found free hydrochloric acid invariably present, and not a trace of any organic acid. In sheep, hydrochloric, associated with varying quantities of lactic acid was found. The latter probably originated from amylaceous food. As long ago as 1824, Prout arrived at the same conclusion from his experiments upon digestion in rabbits. In 1835, Braconnot experimented on animals with a similar result. On the other hand, Bernard and Barreswill, Pelouse and Thompson, attribute the peculiar reaction of gastric juice to lactic acid, the muriatic, according to them, being generated by the decomposition of alkaline chlorides by lactic acid at a high temperature. In 1833, Dr. Dunglison demonstrated the presence of free muriatic acid in large quantities in the gastric juice obtained from the stomach of the well-known Canadian *voyageur* Alexis St. Martin. Quite recently, however, Dr. F. G. Smith had the opportunity of experimenting upon the same individual with the following results. "1st, That the secretions of the stomach when digesting are invariably acid. 2d, That the acid reaction was not due to the presence of phosphoric acid. 3d, That *if* hydrochloric acid was present, it was in very small quantities. 4th, That the main agent in producing the characteristic reaction was lactic acid."* In the stomach of a patient of Dr. Grunewaldt, after abstinence, Dr. Schmidt could detect no free muriatic acid. But in the same person after the stomach was excited by the ingestion of dry peas, hydrochloric acid could be detected in the proportion of 0.1 in 100 parts, while no lactic acid was present.

After some remarks upon the nature of the ferment,—the so called pepsine or gasterase—our author proceeds to consider the action of gastric juice upon nitrogenous food, relying for his conclusions upon the laborious and skilfully conducted experiments of the Professors of the Derpt Laboratory.

From these experiments, it appears that the gastric juice acts upon albuminous matters out of the body, in the same manner as in the stomach, but more slowly and less effectually ; that the free acid alone is not sufficient to accomplish the solution or fer-

* Experiments upon Digestion. By F. G. Smith, M. D. Philadelphia, 1856. See also No. 7, July, 1856, of this Journal.

mentation of albumen; that the digestive power of the gastric juice, is in direct proportion to the acid it contains; that the process of decomposition in aliments impedes their digestion; that the ferment is a soluble substance; that excessive heat destroys the ferment, but low temperatures exert no injurious influence upon it; that when much alkaline saliva is swallowed, the acidity of the gastric juice is more or less diminished with the effect of retarding the digestion of albuminous and increasing that of starchy substances; and finally that an admixture of bile destroys the digestive power of the gastric juice. Dr. Smith, from his experiments upon the influence of human gastric juice upon albuminous, amylaceous and oleaginous aliments, concludes "that gastric juice is a true solvent for animal food; that, in all probability, the observation of Bernard is correct—"that fatty matters undergo no change in the stomach beyond that of disaggregation;" * * * * "that starchy materials are digested in the human stomach; that human gastric juice does not prevent the conversion of starch into grape sugar: and that this conversion may take place in the stomach, independently of the action of saliva, for, as Bernard has shown, any mucous membrane and some alkaline fluids, as the serun of the blood, possess the same power."*

Dr. J. C. Dalton, in commenting upon these experiments of Dr. S., expresses his disbelief in the digestion of amylaceous matters in the stomach, and asserts that "it is extremely probable, to say the least, that the glucose which was detected in the gastric fluids, was not formed during the process of digestion; but *existed beforehand in the bread* used as food."†

In Chapter V. the general and microscopic anatomy of the intestinal epithelium, blood-vessels, lymphatics and glands are noticed.

Formerly the lymphatics were considered the great agents in the absorption of nutritious material from the alimentary canal. Step by step, however, as experimental physiology has advanced, they have been deprived of their importance. First it was pointed out that in the intestines of the invertebrata, where no trace of these organs can be found, digestion and absorption pro-

* Opuscu. cit. p. 16 and Examiner Sept.

† American Journal of Medical Sciences, Oct. 1856, p. 556.

ceeded without difficulty. Then Bernard undertook a series of elaborate experiments, and concluded that the lymphatics were mere absorbers and carrriers of fatty matter, denying to them any agency in conveying albuminous or saccharine matters into the economy. Every physiologist is familiar with his papers in the Comptes Rendus de l' Academie des Sciences, and his published Lectures upon these subjects.

"Dr. Bernard's argument, as supported by his experiments is in short as follows :—

Albumen and sugar require modification before they enter the general circulation ;—

The liver is capable of effecting this modification ;—

Therefore, the liver alone (or almost alone) effects it.

The fallacy lies in the possibility that the required changes may be effected by other means also ; viz., by the digestive juices in the first place ; or secondly, by the intestinal glands during the passage of these alimentary substances through them. Dr. Lehmann's investigations seem to show that the first of these contingencies occurs.

In fine, our present state of knowledge allows us to look upon the bloodvessels and lymphatics as colleagues in the duty of absorption, and as both apparently capable of executing each department in a more or less perfect manner. But the limits of the office of each under ordinary, and still more under varied circumstances, are as yet undefined."

An interesting fact connected with the motion of the chyle along the lacteals is the recent discovery of muscular fibres in the villi.

This chapter concludes with some brief remarks upon a few of the more important points connected with intestinal digestion.

Chapter VI. is devoted to the consideration of the pancreatic juice and its ascertained physiological action. This latter consists according to our author, in its power of converting starch into sugar in the same manner as the saliva—Dr. Bernard's views regarding its agency in the digestion of fat being only partially allowed, for in the *healthy* condition, when "acid gastric juice is mixed with it, the property is lost."

In Chapter VII. the absorptive and blood-making functions of the liver are considered.

"1. The blood of the vena portæ arrives at the liver in a very watery condition, and leaves it much more concentrated. During digestion the water in the portal blood compared with that departing by the hepatic vein is as four to three ; after the completion of digestion, as twelve to five (*Lehmann*). This is a change that was easy to have been anticipated from our knowledge of the great amount of water required for the bile.

2. A more concentrated condition implies a greater proportion of solid matter in the shape of blood globules ; but in addition, it is to be observed that both red and colorless globules are equally increased, and that the former in the hepatic veins are stouter, and less easily destroyed by water than in ordinary blood (*Lehmann.*)

3. The blood of the vena portæ is rich in fat, while that of the hepatic vein has lost this peculiarity. It has come in from the food, and is converted in the liver into part of the bile. Compared with the blood of the general system, the portal blood contains double the amount of fat in the jugular vein (*Lehmann*).

4. In proportion to the blood globules, the albumen is increased in its passage through the liver.

5. The salts are diminished.

6. But the most suggestive of future discovery of all observations on the liver, is that made by Dr. Claude Bernard—namely, that which seems to establish its power of making sugar out of the constituents which the portal blood brings to it.

Dr. Bernard proves his point in the following way :—

1. In man, and in a variety of animals, mammalia, birds, reptiles, fishes, mollusca, experiment proves that the blood of the hepatic veins contains a notable quantity of sugar.

2. The sugar is independent of the quality of the food, and is not derived from that source. For it is found in animals fed exclusively on meat, and in which the blood of the portal system was entirely free from it.

3. A curious circumstance connected with this *hepatic sugar* is the influence of the nervous system over it. Whatever the nature of the food may be, sugar may be made to disappear wholly from the liver by the division of the pneumogastric trunks, or in any way by abolishing their healthy action. Strange to say, an opposite effect—that is to say, an excessive appearance of sugar, an artificial diabetes—was accidentally found to be the result of puncturing a certain spot, accurately defined by the author, in the medulla oblongata, between the trunks of the pneumogastric and acoustic nerves."

Dr. Chambers either has not seen or has forgotten to allude to the able paper on the origin of the sugar found in the liver, read by Figuier at one of the sittings of the Academy of Science, in January 1855. This observer denies entirely the glucogenic function attributed to the liver by Bernard. He thinks that before we can regard the liver as possessing this power of transforming protein compounds into sugar, it is necessary to prove that the saccharine matter contained in the hepatic veins is much greater in quantity than that found in the blood before it enters these veins. To determine this point he instituted, in different animals, a series of analyses of the blood both before and after its passage through the liver. He found .57 per cent of glucose in the blood of a rabbit, while its liver yielded 1 per cent. The blood of the ox contained .48 per cent., that of man .58. His experiments lead him to conclude that "the liver would contain, in equal weight, scarcely twice as much sugar as the blood contained in the other parts of the body," and "that the liver in man and in animals has not received the function of forming sugar, and that all the glucose which it contains in its tissues comes from without, that is to say, from the nourishment." To the depurating office of the liver he attributes the difference in the per centage of saccharine matter, as referred to above. He regards the liver as an organ of condensation and accumulation, a great storehouse, as it were, for glucose, which substance finds its way ultimately to the lungs to undergo that combustive process by which life is at least maintained, if not, indeed, originated.

From the investigations of Drs. Bidder and Schmidt, on the amount of bile secreted by the lower animals in a certain time, we may roughly approximate the amount secreted by man, at three or four pounds per day. Moreover, their observations show that in weakness and disease the amount is diminished; that there is no relation between the amount secreted and the size of the liver; that the secretion appears to be continuous, but is augmented and diminished from time to time according to the stage of digestion; that in cats it reaches its maximum in from ten to twelve hours after the last, full meal, and in dogs, at from 13½ to 15½ hours, and then declines; that a full diet increases not only the absolute quantity of bile, but also the amount of solids; that fatty matter diminishes the flow of the bile; that an animal increases it more than a vegetable diet; and that water increases the quantity within an hour after it is taken, and also to some extent augments the organic solid constituents. According to Hanfield Jones, mercurials, muriate of manganese and colchicum increase the production of yellow matter in the cells of the liver. Nitro-muriatic acid causes a greater discharge of bile per anum. Aloes, oil of turpentine and rhubarb increase the flow of bile into the intestines.

The Derpt Professors have shown that bile, in its natural state, is not capable of dissolving blood-globules; that it will not dissolve coagulated albumen even after prolonged maceration; and furthermore that it does not even assist other juices in digesting albuminoid substances, but influences them as an antiseptic, preventing their putrid decomposition and preserving them safely to be exposed as much as possible to the absorbents of the alimentary canal; that it exerts little or no solvent action over starchy articles of food, but facilitates their regular and orderly digestion by obviating undue acid fermentation; that the great use of the bile is to assist in the digestion and absorption of fatty aliments; and lastly that on its road from the liver to the rectum, the whole of the fluid bile, and at least seven-eighths, and perhaps fourteen-fifteenths, of the solid matter are again taken up by the absorbing vessels.

How the bile enables *neutral* fat to enter the lacteals through a membrane saturated with water is yet undetermined. Bernard, Moleschott, Frerichs, R. and E. H. Wagner, Bidder and Schmidt all differ from each other in their opinions upon this subject.

The uses of the bile in the animal economy may be thus summed up in the words of our author.

"1. To remove from the organism, in common with all other secretions, a small quantity of effete matter.

2. In a manner as yet undetermined, to render oleaginous matters more suitable for being absorbed, or else the membranes more suitable for absorbing them, and that at an advanced period in the course of digestion.

3. To prevent the too rapid decomposition of albuminous food.

4. To form an important part of the great system of circulation through membranes, carrying out substances imperfectly fitted for assimilation, and returning them mixed with new matters introduced by the mouth, into the current of endosmotic absorption."

Passing over chapters VIII and IX, which treat very briefly of the large intestines and succeeding parts, and the gases of the digestive tract, we come to chapter X., which is at once the last, the longest and most interesting of the first book, considering as it does the "physiological action of substances submitted to absorption in the alimentary canal."

"The proper use of all substances submitted to the process of absorption in the alimentary canal may be described as 'nutritive,' in a broad sense—that is to say, to cause the process of nutrition, or the renewal of the tissues which constitutes life, to be carried on in a better way than without them. When instinctively or rationally they are taken by a body in health, with the intention of keeping up that health, they are FOODS; when administered to a sickly body, that is one whose physiological actions are inconvenient to the individual, with the intention of restoring health, they are MEDICINES. Whether an article is food or medicine depends entirely on the intention, and on nothing else.

Both foods and medicines accomplish their objects in one of two ways, in respect of which they may be classed as COMPLEMENTARY or ACCESSORY.

COMPLEMENTARY foods or medicines are those whose nature enables them to become portions of the typical solids or fluids of an animal body. They can rebuild or renew by their own substances those organic compounds which chemical decay is continuously employed in disorganizing.

ACCESSORIES are those by whose use the moulting and renewing (*i. e.* the metamorphosis) of the organic structures are modified, so as best to accommodate themselves to required circumstances. They may be subdivided into those which *arrest*, and those which *increase metamorphosis*, and each subdivision again according to the various tissues upon which their action is mainly exercised. Thus there are arresters and hasteners of metamorphosis of the bones, arresters and hasteners of metamorphosis of blood globules, &c., though in truth we do not yet know enough to assign them all their places."

Section I. of this chapter is taken up with the consideration of Complementary Foods capable of direct absorption without change. These are water, salt, phosphates of soda and potash, carbonate of soda, the sulphates, phosphate and carbonate of lime, and iron. Dr. Chambers also includes oleaginous substances under this head.

Sugar also finds a place in this dietetic class.

Among the Complementary Foods capable of absorption only after previous change by the digestive juices, are starch and albuminous articles.

Under certain circumstances water and common salt become accessory diet. The recent experiments of Böcker, Falck, Bischoff and Boussingault have clearly shown that these articles given in large quantities sensibly increase the metamorphosis of the tissues. The results of the experiments of the first two of these physicians—experiments undertaken to obtain some satisfactory idea of the influence of water on the system—are thus given by the author.

"1. Water increases interstitial metamorphosis or destruction of tissue, and consequent loss of weight.

2. The decomposed tissue is excreted partly by urine and partly in solid fæces.

3. The water formed in the organism by metamorphosis of tissue is augmented, as well as the nitrogenous constituents of the excretions.

4. The excretion of carbon by the lungs, the quickness of pulse or of respiration, is not affected.

5. A necessity for food, as exhibited in the sensation of hunger, keeps pace with the metamorphosis of tissue."

The following judicious remarks on the use and physiological effects of salt, we find on pp. 186–7 :

"The employment of salt in the average healthy state, is decidedly beneficial to the human species, and the use of it as an accessory aliment is wise in those who are well supplied with other food.

"The physiological actions of salt indeed lead us to expect that it must be hurtful in some cases. Where waste is already excessive, or under circumstances where the diet is insufficient, the advantage of salt is a matter of serious doubt. Where food is deficient in quantity or quality, it is evidently improper that any excess of salt should be used beyond that which is just sufficient to act as a complementary aliment; all beyond this increases the waste. Encouragement should be given to employ instead other spicy flavorings which have not this tendency, or which have even a contrary tendency, as we shall presently see reason to believe is the case with several accessory aliments.

"It is to be remarked that the question of the use of salt as an accessory food is by no means the same as that of the employment of salted provisions. The manufacturing process so dries up and hardens the muscular fibre that without diligent cookery it is insoluble in the gastric juice, and in point of fact is an insufficient nutriment, a state of things where it has been said salt is improper. When salted provisions *must* be used, the desideratum is a mode of cookery which would render the albumen and fibrine again soluble."

In the next section, Dr. Chambers proceeds to examine those agents which have been experimentally ascertained to arrest metamorphosis of the tissues, and which, therefore, indirectly or negatively sustain the system, by *preventing* the wear and tear, rather than supplying new material. The most prominent of these is alcohol, an agent which has given rise to not a little discussion among physicians, naturalists and legislators. Here again we are indebted for much valuable information to the skilful and conscientiously scrupulous experiments of Böcker, who arrives at the following conclusions :—

"1. Alcohol diminishes the excretion both of the solid and fluid constituents of the urine. 2. Alcohol does not increase the *cutaneous perspiration* to any practical extent. 3. Alcohol does not augment the *fæcal excretion.* 4. Alcohol diminishes not only the absolute quantity of *carbonic acid exhaled by the lungs,* but also the relative proportion of it in the products of respiration, though it quickens the frequency of the pulmonary movements. It is, therefore, not a supporter of combustion in the body; nay it is a very decided arrester of the oxidation of carbon. 5. The excretion of *water* by the lungs was unaffected to any important extent, that is to say, it was diminished by an amount equal to 1-35th of the whole. This is a further blow to the theory of alcohol supporting combustion."

For a particular account of the author's views upon the dietetic and medicinal use of alcohol, we must refer our readers to the work itself. We shall only observe that great stress is laid upon its prophylactic action against the destructive energies of the mind. By raising the nervous powers also, "it enables a man so to use his body that during the consequent rest he absorbs or fixes enough to place himself in a better state than before."

"It prevents that exhaustion or wearing out of the absorbing tissues which makes the machine less efficient for growth than before; and it allows without damage a certain amount of extra work, bodily or mental, to be imposed, by using indeed the substance of the body for a time, but enabling it to be replaced with interest afterwards."

These advantages have their counterbalancing evil. The question, he remarks, is whether we may not get the good out of it and avoid the evil.

According to the observations of the same careful and industrious German experimentalist above mentioned, the effects of beer on the economy are similar in kind, though less in intensity, than those of pure alcohol. The water combined with the spirit acts as an antagonist to the effects of the latter. The only observed peculiarity was the greater quantity of chloride of sodium excreted in the urine under the use of beer, an effect probably due to one or other of the associated elements, to the extract of hops and of malt, the sugar, ether, &c.

Beer decreases the water of the blood, but increases the fibrine and colored clot. The coagulum on exposure to the air reddens less rapidly than that of normal blood, and contains more pale, non-nucleated globules. Vinous liquors were found by Böcker to diminish the quantity of earthy phosphates in the urine. Sugar, like alcohol, restricts the decomposition of the body, especially of the osseous system. It diminishes not only the whole amount of urine, but the solid and fluid ingredients in different proportions. It has no apparent effect upon the cutaneous perspiration. Contrary to the theory of Liebig, which leads to the classification of sugar as a respiratory or calorifacient element, it is found that this article absolutely tends to diminish the amount of carbonic acid given off from the lungs. In this respect it resembles alcohol, but unlike that substance its effects continue to increase some time after its disuse. It also decreases the amount of watery exhalation from the lungs.

"The earthy phosphates are lessened by more than ½ when sugar is taken, whereas under the use of alcohol the difference was only ⅛th from the average secretion.

"In this remarkable peculiarity in the physiological action of sugar is to be found the reason why it is so largely supplied to the young animal in the mother's milk. The constant motion of the growing limbs demand a rapidity of life in the muscles, and promote a continuous moulting which would be fatal to the stability of bones ; so they are defended against the over-weaning waste by the great quantity of sugar which the mammary secretion always contains. A beautiful provision this is in Nature, but it is still more striking when we find that the supply of sugar to different sorts of animals is in very close proportion as their bones are required to be strong and firm. The lumbering ox, who in his youth staggers about with proverbial ungainliness, and who in really civilized countries is left to only his proper business of getting fat, has the smallest supply of sugar of any commonly known animal.

"These considerations should teach us to be careful not to omit the sugar when we substitute cow's milk for human in feeding our own children, and not to oppose too much the instinctive taste of our young friends for sweets."

The physiological effects of tea, another accessory food, are thus deduced from the experiments of the indefatigable Böcker :

"1. Tea in ordinary doses has not any effect on the amount of carbonic acid expired, the frequency of the respiration or of the pulse. 2. When the diet is insufficient, tea limits very much the loss of weight thereby entailed. 3. When the diet is sufficient, the body is more likely to gain weight when tea is taken than when not. 4. Tea diminishes very much the loss of substance in the shape of urea. 5. Tea lessens remarkably the quantity of fæces secreted. 6. The loss by perspiration is also limited by tea."

For the experiments of Dr. Julius Lehmann upon the effects of coffee on the animal economy, we refer our readers to the January and February Nos. 1854, of this Journal.

We are in want of accurate experiments to determine the physiological effects of chocolate, spices, gums, gelatine, &c. With regard to gelatine Dr. Chambers lays down the following propositions as guides to the practical physician :—

"1. That gelatine is shown by its absence from milk to be unnecessary for the support of the animal body.

2. That, as it is required for the growth of cartilages, sinews, &c., it must be formed from other nitrogenous foods, viz., the albuminoid matters.

3. That as it does not alone prolong animal existence, it cannot replace albuminoid matters, that is, it cannot be converted into them—in other words, albuminoid matter may be turned into gelatine, but gelatine cannot be turned into albuminoid matter.

4. Its failure to prolong life leads to the conjecture that it does not arrest interstitial destruction.

5. However, mixed with other food, it makes it go further, and be more sufficient for the support of life."

Thus much for the Physiology of Digestion. The Second Book opens with a brief, introductory chapter on Health and Disease. Our author's remarks are here of a decidedly philosophical character. He attempts to show that pathology should be regarded as a sort of continuation or modification of physiology, that pathology is merely complementary to physiology, completing the collection of classified facts which constitute the laws of organic life. Such is the argument of the 1st chapter, as shown by the subjoined quotations :—

"It is a fatal error to do or to say anything which can lead to the idea, that in health one set of laws are exhibited by our bodies, and in disease another set; or what is still worse, that these laws are in opposition, the one against the other. * * * * Coma is certainly a diseased condition ; yet it is as much a part of man's nature, as truly physiological that he should be comatose when his brain is pressed upon, as it is that he should be hungry when he has been long without food. * * * * Almost all our patients suffer from the physiological phenomena consequent on a *combination of their special form of a body with external circumstances.* * * * * Disease, therefore, and more especially chronic disease, must be treated not in lots, according to their nomenclature, or as if they were the progeny of some evil power, but according to the mode in which each individual is affected by the union of outward circumstancas with the peculiar form of his body. We must treat the man and not the aliment, or we are as nearly likely to make them better. And we must act with the conviction that everything happens, both as respects the patient's body and the drugs and discipline which we adopt, according to identically the same laws which are to be observed in health. * * * * The important problem for medical science to solve is to know the visible signs and indications of the various combinations of different forms of organs, and their functions, so as to modify, in the way to suit each combination best, the circumstances subject to our will."

To the above views and the therapeutics to which they necessarily lead, we would particularly direct the attention of the profession.

In the II Chapter of this book, Dr. Chambers considers the morbid changes to which the digestive apparatus is liable, observing the same order pursued in describing the normal conditions and functions of these parts.

Morbid alterations of the epithelium of the alimentary canal are comprised under the following heads: Paralysed or Arrested Secretion, The Catarrhal State, Mucous Flux, Excess of Epithelium, The Inflammatory State, The Anæmic State. The physiology and pathology of these conditions, together with some remarks upon their true therapeutical indications and treatment come next in order. Though in some respects rather crudely presented, they are nevertheless well worthy of perusal.

A section is devoted also to a description of the changes in the water circulating through the mucous membrance of the digestive tube.

Our space does not permit any reference to the alterations in the nervous and muscular coats of the stomach, and their consequent morbid functional conditions, nor to the particular changes which the alimentary solvent media undergo in disease.

Chapter III treats of the morbid states of the mouth and gullet which affect digestion—states brought about by mechanical impediments to mastication, impaired salivary secretion, the obscure condition known as cardialgia or heart-burn, &c.

Morbid affections of the stomach constitute the subject matter of Chapter IV.

Mucous flux or chronic gastric catarrh affords us many interesting points of study, especially in its relations to other conditions of the system. The author indicates a number of facts to prove the frequent association of bronchial and gastric catarrh. In this latter disease, milk rendered alkaline by lime-water, being readily absorbed and requiring but little digestive action on the part of the stomach, affords a good diet in the disease. It should be administered in small quantities and frequently. Among other medicinal agents, Dr. C. recommends hydrargyrum cum cretâ, "which, in small doses just sufficient not to act as a purgative, but to promote interstitial destruction, appears to do good." The use of the alkaline sub-salts of soda, especially of the hyposulphite, (in 10 to 15 gr. doses) or a draught of the sulphate of soda with bicarbonate is of decided use in gastric mucous flux, particularly where it is so copious as to produce vomiting. Where the flux is complicated with pyrosis, kino is serviceable. Ipecacuanha is useful by discharging large quantities of mucus. In small and repeated doses, it exerts no beneficial effect. Dr. Chambers discountenances the use of nitrate of silver and of baths in the treatment of this affection, and says that bitters and tonics seldom do good if given before the disorder has received a decided check. Cases of gastric flux kept up by an irritable condition of the nerves of the stomach, and marked by considerable pain, are often relieved by hydrocyanic acid.

Several pages are appropriated to the consideration of the Deficiency of Gastric Juice. This condition is recognised by the following signs:—

"1. An arrest of the food in the stomach. 2. Distress after eating albuminoid substances. 3. Decay of albuminoid substances in the alimentary canal, and consequent fetid gases arising from that decay. 4. The appearance of unaltered muscular fibre in the stools."

Among the causes of lessened gastric secretion may be enumerated "anger, excess in alcohol and overabundant meals; want of the natural stimulus, such as occurs during abstinence; the febrile state, fatigue, mental or bodily, strained attention of the mind, grief, exposure to great heat or cold; anæmia, mucous flux and atonic congestion of the stomach."

Our author gives us a hint as to the physiological relation of the retention of the gastric secretion to certain diseases.

"What, then, are the consequences of the retention of the gastric juice? I should be sorry to dogmatize on a subject confessedly so obscure, but looking at the acidity of the secretion, at the complex and varying nature of the acids it contains, and that there are classes of diseases—viz., gout and rheumatism in their Protean forms—distinguished by the presence of acid in the sweat, the flesh, the blood, and everywhere where it should not be,—that the gastric juice is a peculiarly colorless secretion, and that what is retained in gout and rheumatism is also remarkably devoid of pigment, as is shown by the clear complexions and red blood of the sufferers,—that in gouty and rheumatic persons such deficient digestion as I have described is peculiarly prevalent, and that an attack of these disorders is often preceded by aggravation of the gastric symptoms,—taking, I say, all these things together, I cannot but connect the morbid physiology of these general disorders with the special retention of the secretions of the stomach, and their inability to be elsewhere excreted."

The author's suggestions upon the dietetic and medicinal treatment of this affection, deserve and will repay a careful perusal, while the remarks on degeneration of the glands of the stomach, form not the least interesting portion of this chapter.

But we have already transgressed the limits assigned to this notice, and must, therefore, hasten to conclude, by simply stating that, in the remainder of this chapter, Dr. Chambers considers in detail Ulcer of the Stomach, Cancer and other Mechanical Impediments, Dilated Stomach, Muscular Atony and Vomiting, Imperfect Gastric Digestion, Pain in the Epigastrium, Painful Digestion and Spasmodic Pain. Morbid States of the Intestines receive attention in chapter IV, under the different heads of Defective Absorption, Chronic Ulceration, Mucous Flux of the Intestines, Intestinal Struma, and Defective Excretion. Morbid States of the Pancreas, Liver and Colon occupy the next three chapters. Chapter IX is devoted to the consideration of that very annoying affection—Flatulence. Under the head of Regimen, we find in the concluding chapter (X), some excellent observations upon Spare Diet as a Remedy, The Action of Neutral Salts on the System, Oleaginous Remedies, The Use of Alcohol by Invalids, Obesity as a Consequence of Imperfect Digestion, The General Hygiene of Chronically Deranged Digestion, Use of Insoluble Matters in the food.

We have thus given our readers a rapid glance at the more important points of this excellent work, preferring rather to analyse than to criticise its contents, although in turning over the pages, we have here and there encountered opinions against which we might have opposed some cogent objections. The chief merit of the volume, as will have been seen from the foregoing *resumé*, is its earnest inculcation of a *strictly scientific method* in medicine —the application of the static and dynamic laws of health or normal life to the management and amelioration of disease or abnormal life. "In this," to use the language of our author, "lies the chief hope of continuing the recent advance of our art. The suggestions of physiology must indeed be confirmed and corrected by experience, and the mind must be kept liberally open to the teachings of that daily mistress; but the positions of the two must not be reversed; nor must either be put out of sight, as is done by those who would base treatment on empirical study alone. One is a bridle and the other is a spur, and to use one without the other is as dangerous as mere routine or a universal panacea; but united and in their proper places, they conduct, I believe, to correct principles, and I know to successful practice."

---

*On the Diseases of Infants and Children.* By FLEETWOOD CHURCHILL, M. D., M. R. I. A., &c. *Second American Edition, enlarged and revised by the author. Edited, with additions,* by WILLIAM V. KEATING, M. D., A. M., &c. Philadelphia. Blanchard & Lea. 1856.

This is, in many respects, a very excellent treatise—displaying throughout its pages a very extended and intimate acquaintance with the labors of previous and cotemporary writers on the subjects it treats of. The bibliographical index at the end of the work contains a list of authors and writers referred to, which covers nine pages. The slightest examination must convince any one how largely these authorities have been consulted, as there is hardly a page in it that does not testify to the fact by its frequent references to this or that American, English or Continental writer. Highly as we prize such research and information, our appreciation of the merits of the work would be much increased, had the author afforded us more of the fruits of his own personal experience. From the advantages he has enjoyed, this must have been considerable; we cannot avoid the expression of our regret, therefore, that so large a portion of the book is taken up with the opinions and statements of others, to the exclusion of that more immediate knowledge which he must have gained, and which no one was better qualified to impart. A comparison of the work with that of Dr. West on the same subject will demonstrate the superiority of the latter in this respect. Notwithstanding this defect in the work, which we are satisfied must have arisen more from an error of judgment in the writer than from any other cause, we consider it to be a very valuable addition to the literature on diseases of children.

In preparing the present edition, the author informs his reader that he has endeavored to gain all the information attainable at the time of its writing. The amount of new matter added is considerable, being manifested by the increase of a hundred pages in the volume, notwithstanding an enlargement in the size of the page. To adapt the work to the wants of American practitioners, for whom, at the solicitation of the publishers, it was written, and to whom the author offers his thanks for the welcome they have given to it, as well as his other volumes, he has incorporated in its pages a large amount of material drawn from the writings of our highest authorities. Grateful as the profession in our country must be to the accomplished author for the trouble he has thus assumed to himself by his endeavors to produce a work "that would prove useful and acceptable to them," we cannot overcome a feeling of mortification that it should have been considered necessary by any one to solicit from a foreign writer that experience and instruction which our own country could have very readily and ably furnished.

In perusing the chapter on Pertussis, we were surprised to find no mention made of what has been shown to be its most frequent complication, and to which we are satisfied the greatest part of the mortality following it is owing. We allude to collapse of the lung. "Simple hooping-cough is rarely fatal," says the author, "and yet the mortality in hooping-cough is very great, arising from the liability of other organs to take on morbid action." The most frequent and fatal of all its complications is then stated to be bronchitis or pneumonia, for which the treatment directed by the author is strongly antiphlogistic—bleeding, leeching, tartar emetic, &c. Now it is well known that true pneumonia, as a pathological condition in the lungs of children under six years of age, is exceedingly rare, while collapse of the lung (the lobular pneumonia of some writers) is very frequent. This fact, the researches of Legendre and Bailly, Drs. Alderson, Gerhard, Rilliet & Barthez and others have distinctly shown. This collapsed condition of the lung is always preceded and accompanied by catarrhal inflammation of the bronchial tubes, and is most probably dependent on it. Its chief symtoms are quickened and imperfect respiration, frequent and small pulse, dulness of percussion in certain regions of the chest, often coming on suddenly, and according to Dr. Rees, in place of expansion, a falling in of certain portions of the thorax.

The importance of the recognition of this pathological condition in its bearing upon treatment, which should be supporting, and in some cases even stimulating, instead of antiphlogistic, must be apparent to every one. That it is by far the most common and dangerous complication of hooping-cough, we have positive evidence in the post-mortem observations of the affection made by Mr. Grailly Hewitt, of London. In a valuable paper read before the Harveian Society, in May 1855, he gives the results of nineteen deaths from hooping-cough, the greater number of which occurred at the St. Marylebone Infirmary. The chief lesion found was collapse of the lung; all the cases in fact, excepting one, in whic hextensive tuberculization existed, presented a greater or less amount of it.

The duties of the American Editor have been chiefly confined to the revision of the press, the difficulty of which was much increased by the fact that a large portion of the work was in MS. His principal addition consists of some interesting remarks upon Hydrencephaloid, the substance of which, however, is given by the author in Chapter III. pp. 91, 2, 3 and 4.

The style in which the work is got up is very creditable to the publishers.

---

*New Elements of Operative Surgery.* By ALF. A. L. M. VELPEAU, Professor of Surgical Clinique of the Faculty of Medicine of Paris, Surgeon of the Hospital of La Charité, &c., *Carefully revised, entirely remodelled, and augmented with a Treatise on Minor Surgery, illustrated by over two hundred engravings, incorporated with the text. Accompanied with an Atlas in quarto of twenty-two plates, representing the principal operative processes, surgical instruments, &c. Translated with additions* by P. S. TOWNSEND, M. D., late Physician to the Seaman's Retreat, Staten Island, New York. *Under the supervision of, and with notes and observations* by VALENTINE MOTT, M. D., Professor of the Operations of Surgery with Surgical and Pathological Anatomy in the University of New York, &c. *Fourth Edition, with additions,* by GEO. C. BLACKMAN, M. D., Professor of Surgery in the Medical

College of Ohio, Surgeon to the Commercial Hospital, &c. In three volumes. New York, Samuel S. & W. Wood, No. 389 Broadway. 1856.

The above work is so well known to the profession that it is unnecessary for us to present any very detailed account of its contents. The first American Edition was published in 1844, and the present edition is the fourth. When we consider that it contains nearly 3000 pages of matter, and that it cannot be called a text-book for the schools, the demand for another edition is strong evidence of the general appreciation of its value. It proves, in fact, that the tendency to compress medical knowledge into manuals, to squeeze the Practice of Medicine or the Practice of Surgery into one volume, has not entirely destroyed the public desire for real storehouses of material. That an encyclopedic work, rich in precepts, facts and experiences as the present is should be in such demand, is most encouraging.

Dr. Blackman, the editor, is entitled to great credit for the valuable contributions, he has incorporated into it. Upon the subjects of operations on the Arteries, of Amputations, and of Exsections of Bones, important additions will be found throughout the work. At the end of the third volume an appendix is added containing many important American contributions which cannot fail to enhance the value of this great work as one of reference.

The typography is good and the publication in every respect creditable to those connected with it.

---

1. *Essays on Infant Therapeutics: to which are added, Observations on Ergot; History of the Origin of the use of Mercury in Inflammatory Complaints; together with the Statistics of the deaths from poisoning in New York in the years* 1841–2–3. By John B. Beck, M. D., Professor of Materia Medica and Medical Jurisprudence in the College of Physicians and Surgeons of the University of the State of New York, &c. &c. Second Edition, enlarged and revised. New York, Samuel S. & William Wood. 12 mo. 1855. pp. 158.
2. *Lectures on Materia Medica and Therapeutics, delivered in the College of Physicians and Surgeons of the University of*

*the State of New York.* By JOHN B. BECK, M. D., late Professor of Materia Medica and Medical Jurisprudence. Prepared for the press by his friend C. R. Gilman, M. D., Professor of Obstetrics, &c., in the College of Physicians and Surgeons, New York. Second Edition. New York, S. S. & W. Wood. 1856. 8vo. pp. 559.

The name alone of the Author of the two works above announced would prove an amply sufficient passport with most American readers. The works themselves, however, have long since won their own more substantial reputation in the first editions, which were published in 1851.

But little remains, therefore, to be said, in chronicling the appearance of the new editions, beyond the expression of our gratification at the manner in which the early exhaustion of the previous issues has verified the verdict which was so generally rendered in their favor by the journals of the day. We are sincerely glad to find that they have been so truly appreciated by the profession of our country; and we doubt not that in their new forms they will continue to enjoy the same measure of success.

The sterling character of many of the practical lessons in therapeutics which they convey, and the convincing weight of authority which the personal reputation of their honored and lamented author impresses on these lessons, renders both essays and lectures peculiarly valuable in their influence upon students and young practitioners. We cordially recommend the volumes, not only as especially safe in their teachings, but as interesting mementoes of one of the foremost and worthiest of the pioneers of American Medical instruction, whose fame and example in authorship and professional acquirement belong to the whole history of our country's scientific progress. As such we think they should be faithfully sought for and treasured by all who have the true interests of that progress in their hearts.

